# Efficiency of chewable toothbrush in reduction of dental plaque in students

**DOI:** 10.1186/s12903-019-0748-y

**Published:** 2019-04-18

**Authors:** Rasa Mladenovic, Andrijana Cvetkovic, Brankica Martinovic, Kristina Mladenovic, Milan Zivkovic, Zoran Arsic, Sasa Mladenovic, Tanja Zecevic Lukovic, Dragana Dakovic

**Affiliations:** 1 Faculty of Medicine, University of Pristina, Kosovska Mitrovica, Serbia; 2 Faculty of Medical Sciences, University of Kragujevac, Kragujevac, Serbia; 3 Faculty of Medicine of the Military Medical Academy, University of Defence, Belgrade, Serbia

**Keywords:** Oral health, Dental plaque, Chewable toothbrush, E-questionnaire, Students

## Abstract

**Background:**

Besides classical and electrical toothbrushes market offers tooth brushes that can be chewed, like chewing gums. The aim of this study was to show the effectiveness of chewable toothbrush versus a conventional brush in the students’ population.

**Methods:**

The prospective study included 346 students. For this research, we used a e-questionnaire for “smart” phones, that students completed outside the dental office. Respondents are divided into two groups: control group used conventional toothbrushes, respondents from the tested group used chewable toothbrush. For assessment of accumulation of the plaque we used TQHI index. For testing statistical hypotheses, the following were used: t-test for two independent samples and analysis of the variance of repeated measurements.

**Results:**

Before brushing teeth, the average TQHI value for chewable brushes is 2.8 ± 0.3, while conventional is 2.7 ± 0.3, which is not a statistically significant difference (*p* = 0.448). After brushing teeth, the average TQHI value for chewable brushes is 2.0 ± 0.1, while conventional 2.0 ± 0.3, which is also not statistically significant (*p* = 0.729). Observing the index of the plaque values on the tooth surfaces in the upper jaw, in both groups, there was a statistically significant change in the amount of plaque in time (*p* < 0.001). There is a statistically significant interaction between groups and changes in the amount of plaque in the observed period (*p* = 0.013).

**Conclusions:**

The fact that there is no significant difference in the effectiveness of the tested brushes indicates the benefits of using chewable toothbrushes in order to reduce plaque, primarily in the inability to use conventional brushes.

## Background

Dental plaque, also known as dental biofilm, represents a non-mineral community of microorganisms organized in the organic mucopolysaccharide matrix and is the main etiological factor for the formation of caries and periodontal diseases [1].

Maintaining oral hygiene is an important factor in the fight against dental caries and periodontal diseases, especially for urban and civilized people, who consume refined foods. The level of oral hygiene affects the microbial accumulation on the teeth, and the control of deposits is the basis for the prevention of oral diseases [2].

Numerous studies have shown that there is a positive correlation between level of education and oral health status [3, 4]. Therefore, students represent an important group of population that can be examined to assess oral health, awareness and habits among young people and educated groups [5].

According to the recommendations of the oral health protocol, it is ideal to brush your teeth twice a day for continuous care [6, 7], whereas a conventional toothbrush can be as effective as an electric brush with the proper brushing technique [8, 9].

In addition to conventional and electric toothbrushes, chewable, toothbrushes that are chewed like a chewing gum can also be found on the market. Some of them contain a very high concentration of Xylitol (95%) - a natural sweetener that is very effective in preventing the formation of caries [10].

The aim of this study was to compare the effectiveness of chewable toothbrush and a conventional brush in the student population..

## Methods

This prospective study involved 346 students from the University of Pristina, aged between 21 and 24 (average age 23 years). The study was approved by the Ethical Committee and written consents were obtained from all subjects. Clinical examinations were conducted at the University Clinic of Dentistry, Falulty of Medicine, University of Pristina, located in Kosovska Mitrovica. The examinations were conducted during lunch time (12.00–14.00 h). All examinations were performed by a single examiner.

### Questionnaire

“Oral Health Questionnaire” for students was created as an application for ‘smart’ phones with Android platform, the developer being the corrensponding author. Students of the University of Pristina were informed that the “Dent.IN TEST” application could be downloaded free of charge from the Google Play Store.

**Fig. 1:**
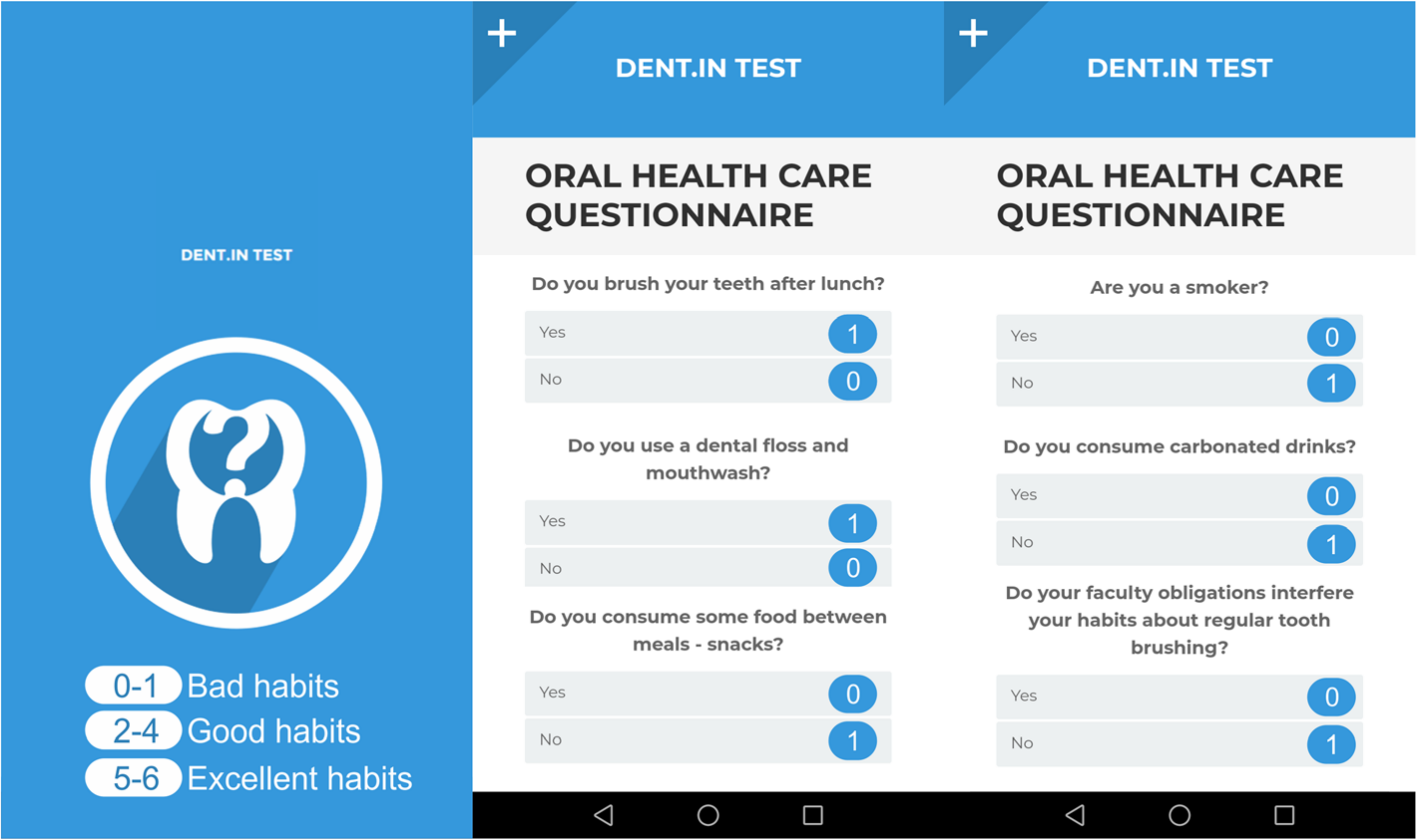
Android application questionnaire ‘Dent.in TEST’ and scoring

**Fig. 2:**
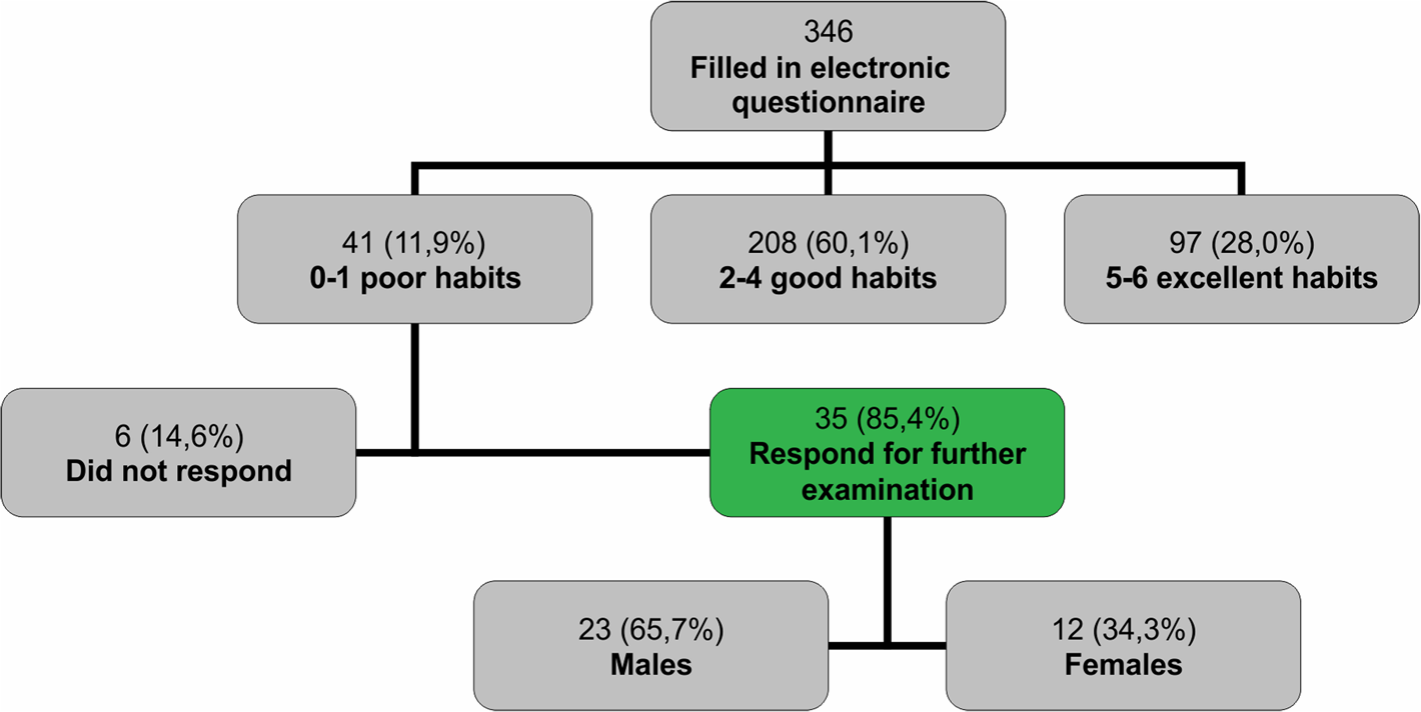
Research protocol

The questionnaire contained information on poor oral hygiene practices, and each question scored one point (Fig. 1). The ‘Dent.in TEST’ application recorded and classified data from all subjects, whereas the students with the score of 0–1 Poor dental care were automatically scheduled for clinical examination using the contact they provided at the end of the questionnaire. Of the 346 students who completed the e-questionnaire, 41 (11.8%) had a score of 0–1 Poor dental care. 6 of these (14.6%) did not report for clinical examination, thus a total of 35 students (85, 4%) were included in further testing (Fig. 2 and Table 1).

**Table 1:** Gender distribution of students

Sex	Control group (n)	Test group (n)
Male	13	10
Female	4	8

### Clinical examination

To identify dental plaque, we used disclosing tablets (TePe® Plaq-Search Disclosing Tablets), and to estimate the plaque accumulation TQHI index (Turesky modification of the Quigley-Hein Index) [11, 12]. The TQHI index evaluates the plaque revealed on the buccal and lingual non-restored surfaces of the teeth. An index for the entire mouth is determined by dividing the total score by the number of surfaces examined.

Control group - 17 subjects: Control subjects tested the TePe® Select ExtraSoft toothbrushes and toothpaste. Plaque index values were recorded before and after 2- min -brushing in the manner to which they were accustomed. No mirror was available during the brushing procedure.

**Fig. 3:**
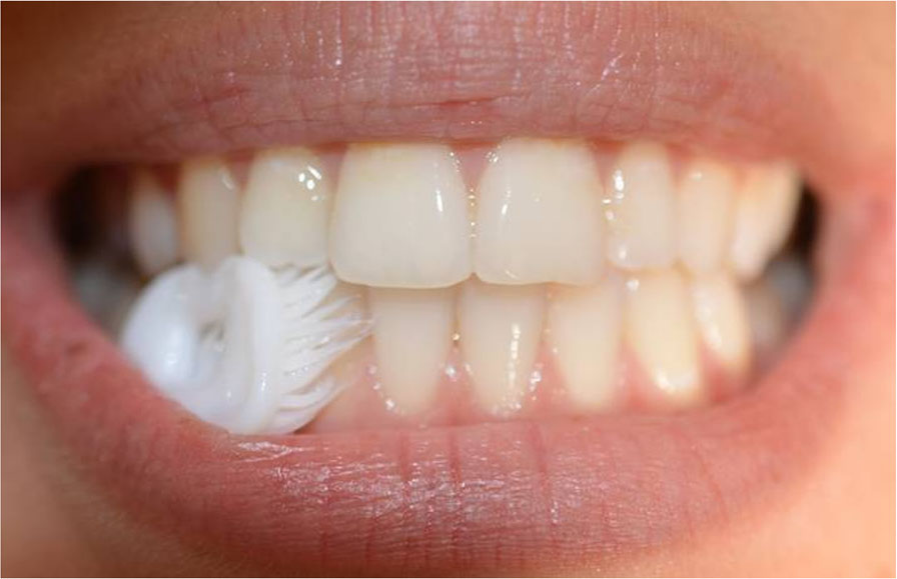
“Fuzzy Brush” chewable toothbrush

Test group - 18 subjects: Subjects from the tested group used chewable ‘Fuzzy brush’ (Fuzzy Brush Ltd., London, UK) approved by the US Food and Drug Administration. It is a unique ‘all in one’ chewable toothbrush and breath freshener, designed by dentists. No toothpaste or water is required and it contains high concentrations of Xylitol (Fig. 3). Students were instructed on how to use the chewable brush for 3 min, in line with manufacturer’s recommendations. Plaque index values were recorded before and after tooth brushing.

### Statistical methods

For the analysis of primary data, descriptive statistical methods and methods for testing statistical hypotheses were used. Of the descriptive statistical methods, the measures of central tendency (arithmetic mean), mea surements of variability (standard deviation) and relative numbers (structural indicators) were used. Of the methods for testing statistical hypotheses, the following were used: t-test for two independent samples and the repeated measurements analysis of variance. Statistical hypotheses were tested at the level of statistical significance (alpha level) of 0.05.

## Results

Before brushing, the average TQHI value for Fuzzy Brush brushes was 2.8 ± 0.3, while TePe Classic was 2.7 ± 0.3, which was not a statistically significant difference (*p* = 0.448). After brushing, the average TQHI value for Fuzzy Brush brushes was 2.0 ± 0.1, while TePe Classic 2.0 ± 0.3, which was also not statistically significant (*p* = 0.729) (Table 2).

**Table 2:** TQHI score before and after brushing

Tooth surface		Chewable toothbrush	Conventional toothbrush	*p*-value
Upper jaw	Before brushing	2.5 ± 0.3	2.4 ± 0.3	0.437
	After brushing	1.7 ± 0.3	2.0 ± 0.4	0.024
Lower jaw	Before brushing	2.9 ± 0.4	2.8 ± 0.4	0.548
	After brushing	2.3 ± 0.3	1.9 ± 0.5	0.008
Front teeth	Before brushing	2.8 ± 0.5	2.7 ± 0.5	0.771
	After brushing	2.0 ± 0.3	2.0 ± 0.5	0.720
Lateral teeth	Before brushing	2.7 ± 0.5	2.6 ± 0.5	0.664
	After brushing	1.9 ± 0.3	2.0 ± 0.4	0.313
Oral surface	Before brushing	2.9 ± 0.5	2.9 ± 0.5	0.619
	After brushing	2.3 ± 0.3	2.5 ± 0.5	0.197
Buccal surface	Before brushing	2.6 ± 0.5	2.5 ± 0.5	0.574
	After brushing	1.7 ± 0.4	1.5 ± 0.3	0.209
All teeth surfaces	Before brushing	2.8 ± 0.3	2.7 ± 0.3	0.448
	After brushing	2.0 ± 0.1	2.0 ± 0.3	0.729

**Fig. 4:**
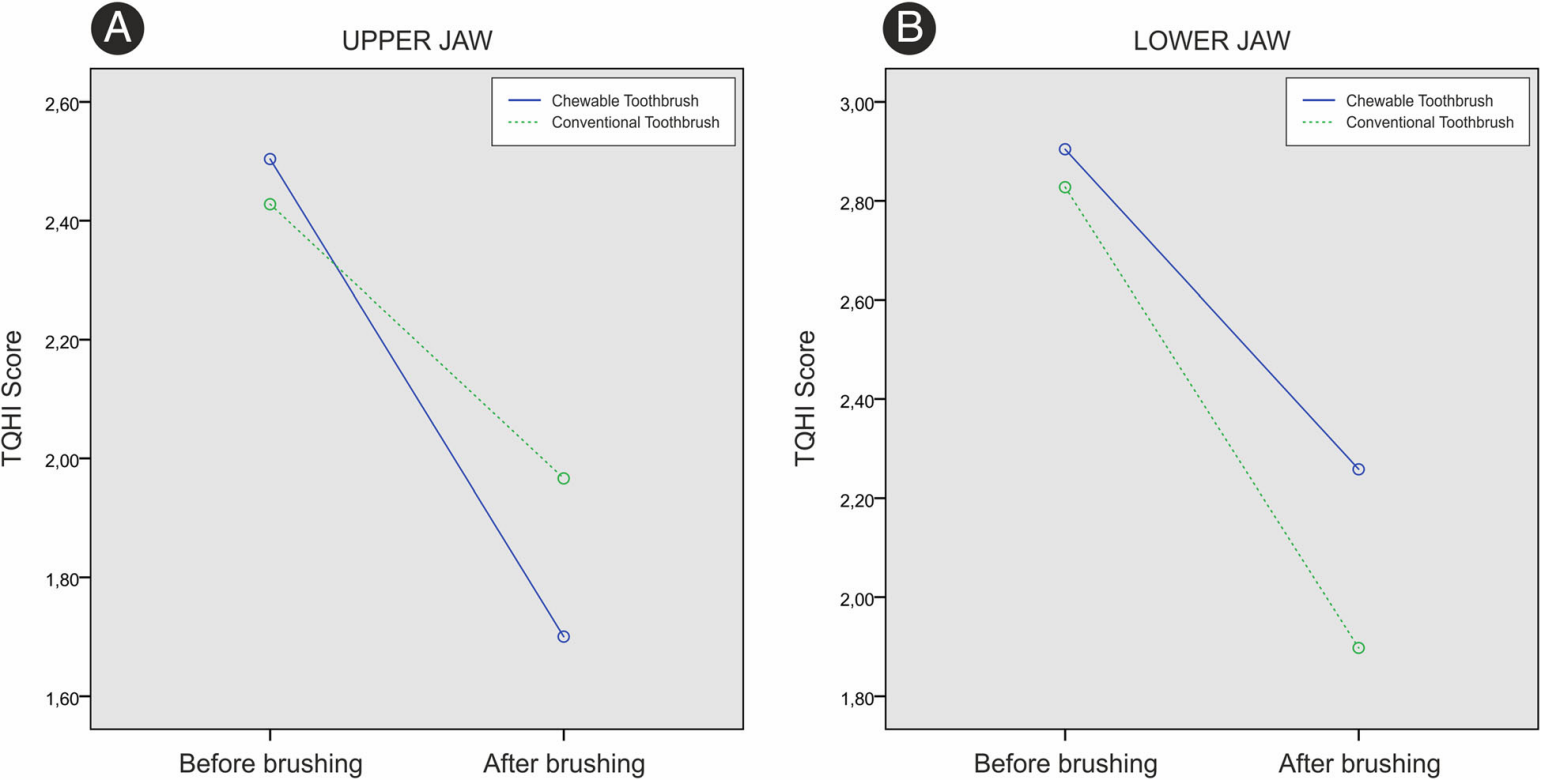
**a** TQHI score in upper jaw, **b** lower jaw

Observing plaque index values for the tooth surfaces in the upper jaw, there was a statistically significant change in the plaque volume over time in both groups (*p* < 0,001). In the observed period, there was no statistically significant difference in plaque index values in examined toothbrushes (*p* = 0.254). The intergroup comparison revealed statistically significant change in plaque volume in the observed period (*p* = 0.013) (Fig. 4a).

Overall observation of the plaque index values on the tooth surfaces in the lower jaw reveals a statistically significant change in plaque volume over time in both groups (*p* < 0.001). There was a statistically significant difference between groups in plaque volume values (*p* = 0.002), however, intergroup comparison revealed no change in plaque volume values in the observed period (*p* = 0.198) (Fig. 4b).

**Fig. 5:**
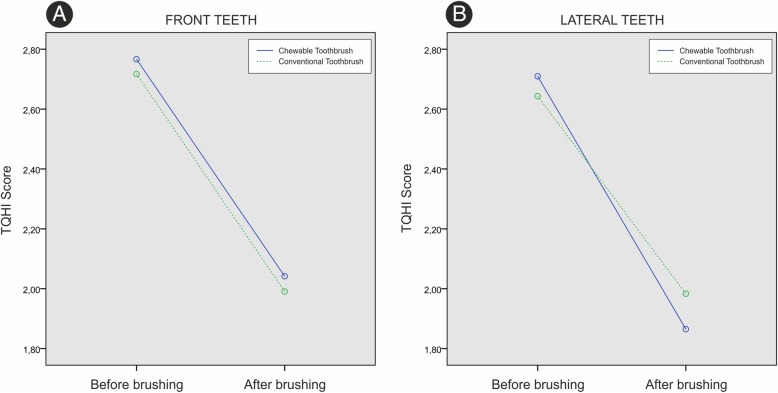
**a** TQHI score in front teeth, **b** lateral teeth

Regarding the plaque index measured on the front teeth, there was a statistically significant change in plaque volume over time in both groups (*p* < 0.001). In the observed period there was no statistically significant difference in plaque volume values (*p* = 0.684). The intergoup comparison revealed no statistically significant change in plaque volume values in the observed period (*p* = 0.993) (Fig. 5a).

In the case of lateral teeth, there was a statistically significant change in the amount of plaque in time in both groups (*p* < 0.001). Overall, there was no statistically significant difference in plaque volume values between the groups (*p* = 0.822); intergroup comparison revealed no change in plaque volume values in the observed period (*p* = 0.191) (Fig. 5b).

**Fig. 6:**
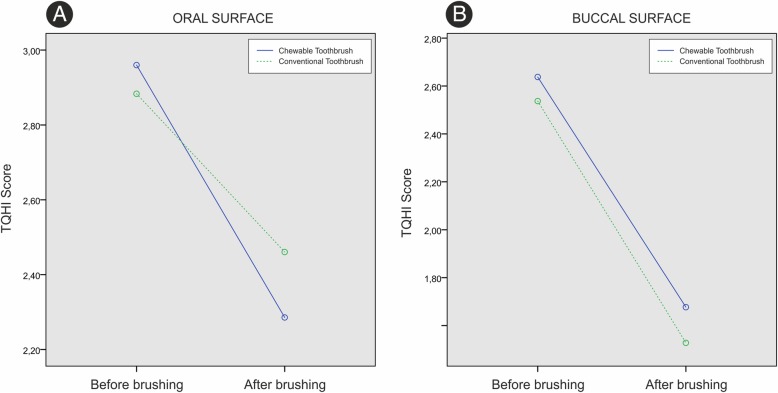
**a** TQHI score of oral surface, **b** buccal surface

In the oral teeth surfaces, there was a statistically significant change in plaque volume over time in both groups (*p* < 0.001). Overall, there was no statistically significant difference in plaque volume values between the groups in the observed period (*p* = 0.685); intergroup comparison for plaque volume in the observed period did not reveal significant statistical change (*p* = 0.107) (Fig. 6a).

Concerning the buccal surfaces of the teeth, there was statistically significant change in plaque volume values over time (*p* < 0.001). In the observed period, there was no statistically significant difference in plaque values between the groups (*p* = 0.284). The intergroup comparison for plaque volume in the observed period did not reveal significant statistical change (*p* = 0.804) (Fig. 6b).

**Fig. 7:**
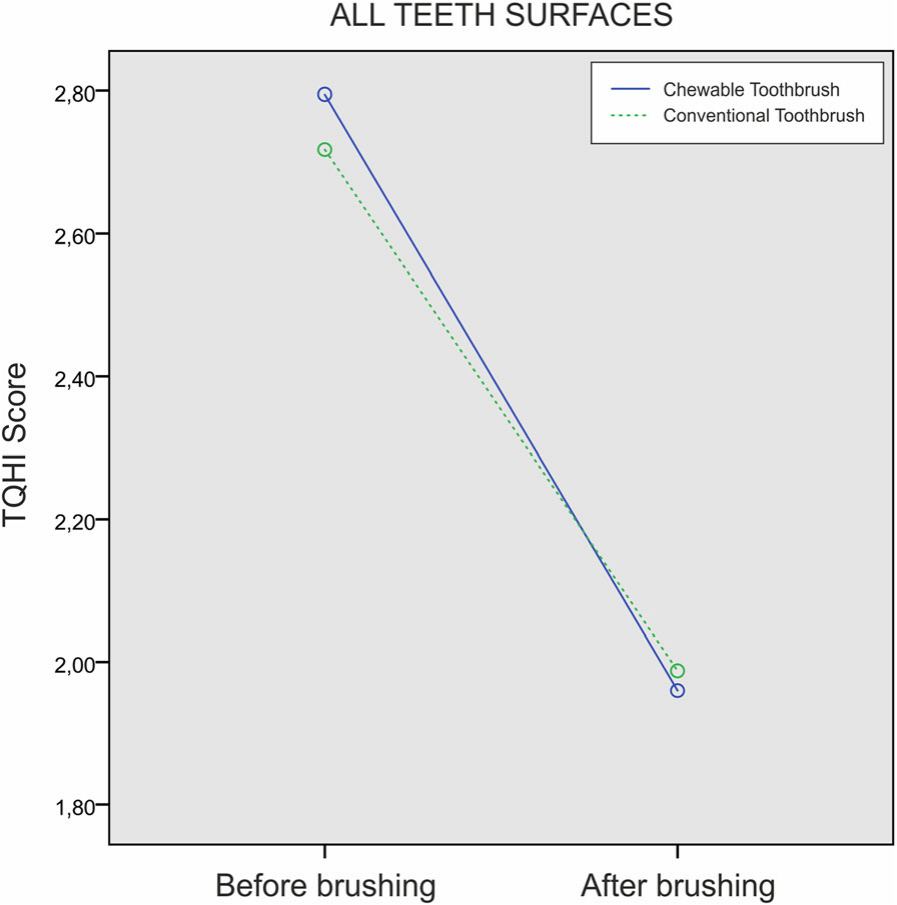
TQHI score of all teeth surfaces

Observing all teeth surfaces included in the TQHI index, there was a statistically significant change in plaque volume over time in both groups (*p* < 0.001). In the observed period, there was no statistically significant difference in plaque volume values between the groups (*p* = 0.708). The intergroup comparison for plaque volume in the observed period did not reveal significant statistical change (*p* = 0.408) (Fig. 7).

## Discussion

The ability of patients to properly and thoroughly clean the oral cavity depends to a great extent on their technique, the time of brushing, and the type of brushes used. When choosing a brush, there are a number of ergonomic elements that can influence the brush type selection. In the era of modern and ‘fast’ lifestyles, besides the design of the head, shape and angle of the bristles, an important ergonomic element is certainly the ease of use and availability. In this context, this study dealt with a comparative analysis of the degree of dental plaque reduction in subpopulation of students.

Comparing the ‘Fuzzy Brush’ brushes with conventional ones, we noticed a statistically significant difference between the upper and the lower jaw teeth surface. The ‘Fuzzy Brush’ brush effectively removed the plaque on the tooth surfaces in the upper jaw compared to the control brush. The values of plaque indices after toothbrushing are nevertheless high in both brushes. In support of this, no mirror was available during brushing procedure with conventional brush. Also, according to the recommendations of the chewing brush manufacturer, chewing exercise is necessary, and 2–3 uses are required for the best effect, whereas in the study we used it once. No studies were conducted on the above comparison, thus we can not compare the obtained values.

In studies published by Myoken et al. [13] and Bezgin et al. [14], Fuzzy brush was more effective in reducing tooth plaque from oral surfaces of the teeth in children compared to a conventional brush, while in our study no statistically significant difference was observed. Our study has shown that the use of both brushes leads to a significant reduction in plaque on all dental surfaces. Although there are few studies on this topic, the obtained results of our work are fully in line with the ones conducted so far [13–15]. All this confirms the initial hypothesis that ‘Fuzzy Brush’ brushes can be an alternative to the use of conventional brushes, especially in situations of inability of their application. Bearing in mind that high concentration of Xylitol inhibits the growth of mutans streptococci in the dental plaque, a more extensive study is required to evaluate the impact of long-term use of the chewable brush in reduction of caries incidence [16, 17]. Certain studies state that the use of chewing gum can relieve stress and depression, which is yet another attribute of the chewable brush [18, 19].

Although this study included only the student population, for ease of use, we consider that ‘Fuzzy Brush Brushes’ can be especially useful in the maintenance and improvement of oral health in people with special needs, in elderly with motor skills difficulties and in patients with more severe degrees of musculoskeletal system impairments. Also, chewing is a common behavior in special needs children, especially those with autism or ADHD (Attention deficit hyperactivity disorder). Children with sensory issues often feel compelled to chew on paper, clothes, or other objects. This hypothesis can be the starting point for future studies on the effectiveness of examined brushes in the preservation of oral health in children with autism or ADHD.

The use of a smartphone is growing day by day for both personal and professional purposes. They are becoming a more suitable tool for improving education in developing countries and become popular as an effective educational tool [20–22]. For the purpose of the study a questionnaire was made in the form of an application for smartphones (Dent.IN TEST App) which students completed outside the dental office. The application automatically classified students according to the oral hygiene practices, and students with poor practices were contacted for further research. This type of questionnaire is simpler, less demanding and more economical in terms of time management, and the special convenience of using a smart phone for educational purposes is to enable continuous communication and interaction between participants and examiners [23].

## Conclusions

Our conclusion is that both types of brushes are effective in removing dental plaque. The fact that there is no significant difference in the effectiveness of the tested brushes indicates the benefits of using ‘Fuzzy brush’ brushes in order to reduce plaque, primarily in the inability to use conventional brushes.

## Abbreviations

ADHD: Attention deficit hyperactivity disorder
TQHI: Turesky modification of the Quigley-Hein Index
